# A Novel Model Using AAV9-Cre to Knockout Adult Leydig Cell Gene Expression Reveals a Physiological Role of Glucocorticoid Receptor Signalling in Leydig Cell Function

**DOI:** 10.3390/ijms232315015

**Published:** 2022-11-30

**Authors:** Anne-Louise Gannon, Annalucia L. Darbey, Grace Chensee, Ben M. Lawrence, Liza O’Donnell, Joanna Kelso, Natalie Reed, Shanmathi Parameswaran, Sarah Smith, Lee B. Smith, Diane Rebourcet

**Affiliations:** 1 College of Engineering, Science and Environment, The University of Newcastle, Callaghan, NSW 2308, Australia; 2 MRC Centre for Reproductive Health, The Queen’s Medical Research Institute, University of Edinburgh, 47 Little France Crescent, Edinburgh EH16 4TJ, UK; 3 Office for Research, Griffith University, Southport, QLD 4222, Australia

**Keywords:** glucocorticoid receptor, androgens, leydig cells, AAV9, steroidogenesis

## Abstract

Glucocorticoids are steroids involved in key physiological processes such as development, metabolism, inflammatory and stress responses and are mostly used exogenously as medications to treat various inflammation-based conditions. They act via the glucocorticoid receptor (GR) expressed in most cells. Exogenous glucocorticoids can negatively impact the function of the Leydig cells in the testis, leading to decreased androgen production. However, endogenous glucocorticoids are produced by the adrenal and within the testis, but whether their action on GR in Leydig cells regulates steroidogenesis is unknown. This study aimed to define the role of endogenous GR signalling in adult Leydig cells. We developed and compared two models; an inducible Cre transgene driven by expression of the *Cyp17a1* steroidogenic gene (*Cyp17*-iCre) that depletes GR during development and a viral vector-driven Cre (AAV9-Cre) to deplete GR in adulthood. The delivery of AAV9-Cre ablated GR in adult mouse Leydig cells depleted Leydig cell GR more efficiently than the *Cyp17*-iCre model. Importantly, adult depletion of GR in Leydig cells caused reduced expression of luteinising hormone receptor (*Lhcgr*) and of steroidogenic enzymes required for normal androgen production. These findings reveal that Leydig cell GR signalling plays a physiological role in the testis and highlight that a normal balance of glucocorticoid activity in the testis is important for steroidogenesis.

## 1. Introduction

Stress and illness are known to reduce testosterone production by the testis. Although the mechanisms underpinning this observation remain unclear, it is widely accepted that, under stress conditions, the hypothalamus-pituitary-adrenal axis acts to suppress gonadal function as fertility is a secondary consideration to survival [1]. The production of androgens by the testis is essential for men’s health and fertility. Disruption to the production or action of androgens is associated with lifelong chronic pathologies, including infertility [2,3,4,5].

It is well established that elevated circulating levels of glucocorticoids, such as those induced during inflammation, reduces androgen production and fertility in men [6,7]. Exogenous glucocorticoids are commonly used to treat a wide variety of inflammatory conditions, such as arthritis, asthma, and allergies. Conversely, long term medication or prolonged stress can lead to glucocorticoid resistance, which can also impact on androgen production [6]. Therefore, before such therapies are considered, it is essential to establish the role of GR in the steroidogenic Leydig cells.

Glucocorticoids act via a ligand activated nuclear receptor GR (encoded by the nuclear receptor subfamily 3 group member 1 gene *Nr3c1*). GR signalling regulates the homeostasis between basal and stress-related conditions of key processes such as metabolism and immune functions [8,9,10]. Glucocorticoids can also act at the level of the hypothalamus-gonad axis and stress, or exogenous glucocorticoids can shut down the reproductive function and inhibit LH pulsatile secretion which can lead to a reduction of testosterone production [11]. In the testis, GR is expressed in both somatic and germ cells [12,13,14,15]. GR signalling in Sertoli cells is required for normal Sertoli cell maturation, spermatogenesis and for Leydig cell steroidogenesis [16], suggesting a physiological role of GR in the testis. Whilst most glucocorticoids are produced by the adrenal, resident interstitial macrophages produce glucocorticoids within the testis [17] and the intratesticular concentration of glucocorticoids is tightly regulated by specific metabolizing enzymes (11β-hydroxysteroid dehydrogenase enzymes, or HSD11Bs) [14,18]. Previous studies have shown that elevated glucocorticoids can regulate testicular LH receptor expression [19,20] and decrease testosterone production by Leydig cells via a reduction in the expression of steroidogenic enzymes [21,22,23,24,25]. However, whether GR signalling in Leydig cells supports normal steroidogenesis is unknown.

To investigate the role of GR signalling in Leydig cells, we developed and compared two inducible transgenic mouse models to specifically ablate GR signalling in Leydig cells along testis development; an inducible Cre transgene driven by expression of the *Cyp17a1* steroidogenic gene (*Cyp17*-iCre) and a viral vector-driven Adeno Associated Virus 9 Cre (AAV9-Cre). The results demonstrate that viral-mediated vectors can be used to selectively knockdown gene expression in adult Leydig cells and reveal that GR signalling regulates Leydig cell steroidogenesis. 

## 2. Results

### 2.1. Confirmation of Cyp17-iCre Leydig Cell Cre Recombinase Activity

**Figure 1:**
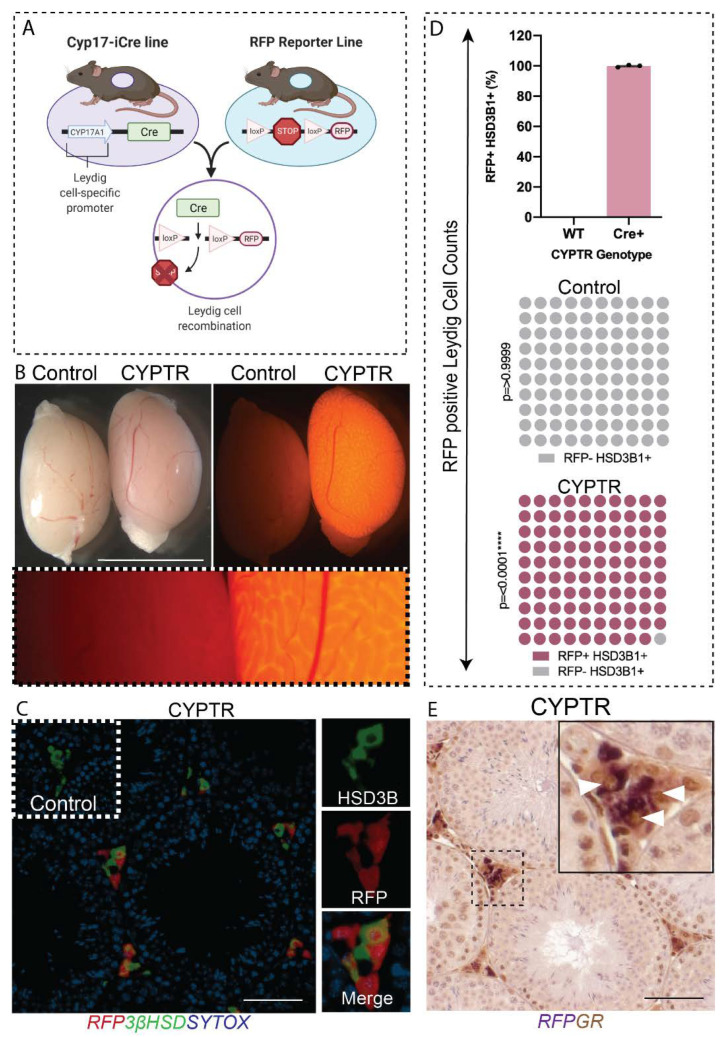
Confirmation of RFP expression in testes from Cyp17a1-iCre:RT26 RFP (CYPTR) mice. (**A**) Diagram of the breeding strategy to generate Cyp17-iCre;RT26 RFP reporter (CYPTR) mice. (**B**) Representative epifluorescence microscopy images of freshly dissected CYPTR testis at d80 confirming RFP expression in adult testis. Organs were imaged under an RFP filter and show a lack of RFP in Controls but positive RFP expression in CYPTR testis (fluorescent images taken after 5.5 s of exposure). Scale bars 0.5 cm. (**C**) Dual-label immunofluorescence of RFP and the testicular Leydig cell marker protein 3βHSD (green), counterstained with the nuclear stain Sytox (blue). Insets demonstrate 40× magnification of single channel 3βHSD, RFP and merged channels. (**D**) Percentage of 3βHSD+ interstitial cells that co-express RFP in wildtype and CYPTR mice, indicating that CYPTR mice express RFP in Leydig cells, whereas wildtype mice do not (*n* = 3 per group, Chi Square *p* = 0.0001). (**E**) Double immunostaining of RFP (purple) and GR (brown) in testis from adult CYPTR mice demonstrated co-localisation of RFP with GR in Leydig cells. Insets demonstrate 40× magnification of single channel 3βHSD, RFP and merged channels. Scale Bars 50 μm.

GR is widely expressed in the testis, including in Leydig cells, Sertoli cells and germ cells from fetal life through to adulthood [12,15,16]. The transgenic mouse line expressing an inducible Cre recombinase (iCre) under the control of the *Cyp17a1* (cytochrome P450 17αhydroxylase/17, 20-lyase) promoter (referred to as *Cyp17*-iCre) [26] was used to direct gene deletions to testicular Leydig cells and ovarian theca cells. The *Cyp17a1*-iCre line was bred to a Rosa-26:RFP reporter floxed line (CYPTR) (Figure 1A). Previous studies using an independently derived line have shown that the transgene is active in E16.5 testes through to adulthood [26]. Epifluorescence analysis of freshly dissected CYPTR mice at day (d) 80 confirms RFP expression in the testis (Figure 1B), but expression was also observed in the adrenal, epididymis and liver (Supplementary Figure S1). Immunohistochemical localisation of RFP and the Leydig cell-specific marker protein 3βHSD in CYPTR testis sections demonstrated co-localisation of RFP and 3βHSD in Leydig cells, with no off-target expression in other testicular cells (Figure 1C). To determine targeting efficiency, we quantified the percentage of Leydig cells (3βHSD-positive cells) that were RFP-positive and showed that 99% of Leydig cells expressed the CYPTR transgene (Figure 1D). Finally, we determined that the *Cyp17*-iCre transgene co-localised with GR positive Leydig cells (Figure 1E). Taken together, these data confirm that the *Cyp17*-iCre transgene is specific to Leydig cells in the testis and co-localises with Leydig cell GR, making it a suitable model for the deletion of GR in Leydig cells. 

### 2.2. Assessment of Glucocorticoid Receptor Ablation in Leydig Cells Using a Traditional Cre/LoxP Model

We next utilised the *Cyp17*-iCre line to generate a mouse model with Leydig cell-specific GR ablation. We bred the *Cyp17*-iCre females to homozygous (Hom) GR^flox/flox^ males (‘GRGR’ mice) [27], resulting in an offspring heterozygous (Het) for Cre^+/^;GR^flox^/^+^ or Cre^−/^;GR^flox^/^+^. For total Leydig cell GR ablation, Cre^+/^; GR^flox^/^+^ males were again bred to Gr^flox/flox^ female resulting in the following offspring: Cre^−/^;GR^flox^/^flox^ (Cre- Hom) termed ‘littermate control’, Cre^−/^;GR^flox^/^+^ (Cre-Het), termed ‘Het littermate control’, Cre^+/^;GR^flox^/^flox^ (Cre+ Hom), termed ‘CYP GR knockout’ (CYPGRKO), Cre^+/^;GR^flox^/^+^ (Cre+ Het), termed ‘Het CYPGR knockout’ (Het CYPGRKO). 

**Figure 2:**
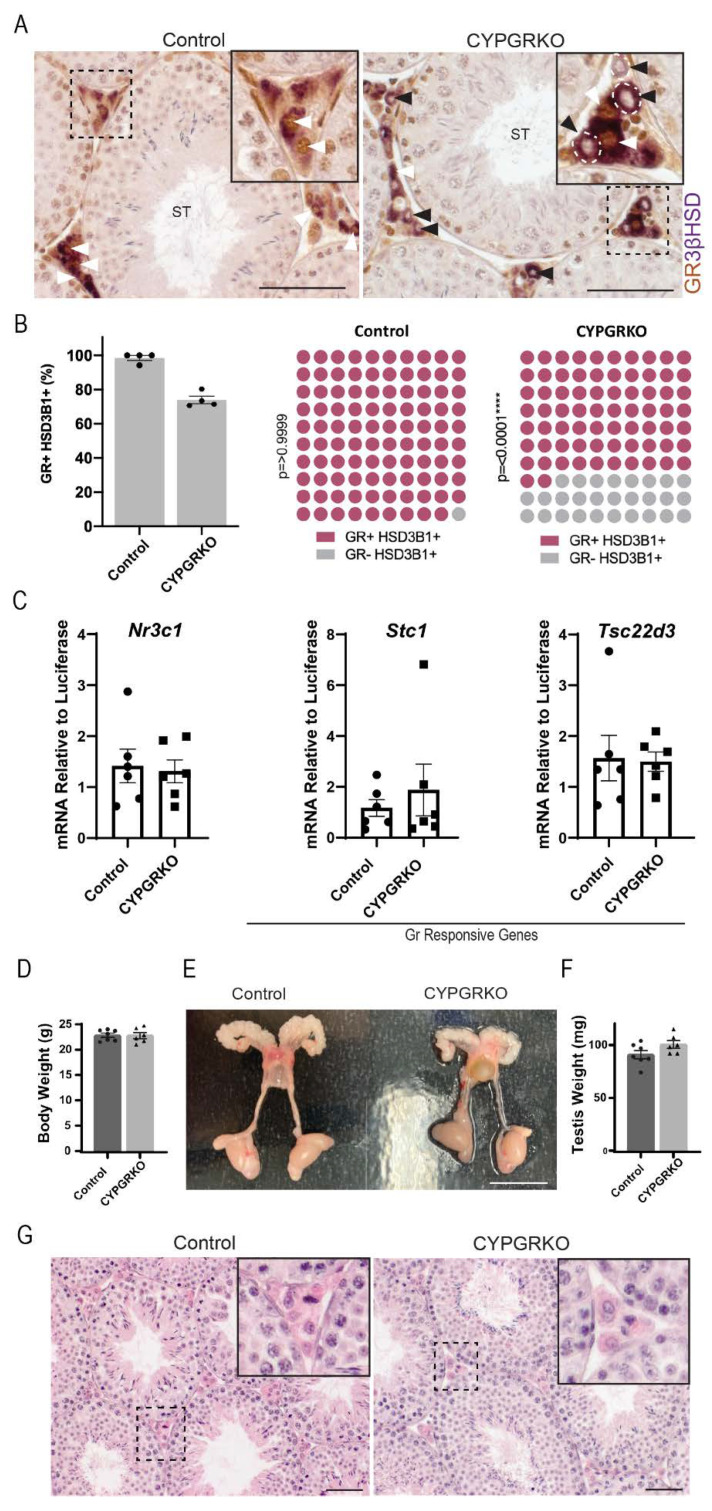
Validation of glucocorticoid receptor ablation in CYPGRKO Leydig cells. (**A**) Immunohistochemical localisation of testicular GR demonstrates ablation in 3βHSD+ Leydig cells but not in other testicular cells. Black arrowheads denote Leydig cells with GR ablation, white arrowheads denote Leydig cells with GR intact. (**B**) Percentage of cells co-expressing GR protein and the Leydig cell marker 3βHSD demonstrates a significant reduction in the number of Leydig cells expressing GR (*n* = 3, Chi Square *p* = 0.0001). (**C**) Comparative analysis of testicular Nr3c1 gene expression shows no changes in CYPGRKO when compared with littermate controls. Direct GR response gene transcripts Stc1 and Tsc22d3 do not show any changes in CYPGRKO when compared with littermate controls (one-way ANOVA; *n* = 6–8, Tukey’s post hoc analysis, error bars SEM). Scale Bars 50 μm. Annotations; I, Interstitium; ST, seminiferous tubule). (**D**) Adult body weight of CYPGRKO compared to control did not change. (**E**) The gross morphology of the reproductive system in adult (d80) was comparable across the different groups (*n* = 6–7, t test). (**F**) No difference was noted in adult testis weight between groups (*n* = 6–7, T Test), and (**G**) testicular histology was normal. Scale Bars 50 μm.

Co-immunohistochemical localisation of GR and 3βHSD in adult (d80) control mice demonstrated that GR is expressed in the nuclei of most interstitial cells; Leydig cells, endothelial cells, and macrophages; as well in the tubules in Sertoli and peritubular myoid cells and early stage germ cells. In contrast, in the CYPGRKO testis, GR depletion was observed in some, but not all, Leydig cells (black and white arrowheads) (Figure 2A). Stereological quantification of GR positive and negative Leydig cells (3βHSD positive cells) in control and CYPGRKO revealed that an average of 28% Leydig cells had lost their GR expression (Figure 2B). *Nr3c1* transcripts and direct downstream targets of GR, *Stc1* and *Tsc22d3* are unperturbed in the CYPGRKO testis in all cohorts analysed (Figure 2C). In adulthood (d80), there was no difference in body weight between the genotype and the gross reproductive system of CYPGRKO males appeared unchanged compared to control (Figure 2D,E). Testis weight remained unchanged, and the histology of the testis showed no differences between control and CYPGRKO males (Figure 2F,G).

Despite the significant decrease in the number of GR-positive, 3βHSD-positive Leydig cells in CYPGRKO mice, no changes were observed in the expression of Leydig cell-enriched steroidogenic enzyme transcripts (Supplementary Figure S2). Leydig cells are well known for their ability to show steroidogenic compensation following physiological challenge or manipulation, and normal steroidogenesis can be observed in mice with as little as 30% functional Leydig cells [28,29]. The results suggest that the targeting efficiency of the *Cyp17*-iCre in Leydig cells is only able to ablate GR in 28% of Leydig cells and this is not sufficient to impact testis function. Thus, CYPGRKO mice are an inadequate model to study the role of GR in Leydig cell development and regulation. 

### 2.3. Validation of the AAV9 Inducible Cre/loxP System

**Figure 3:**
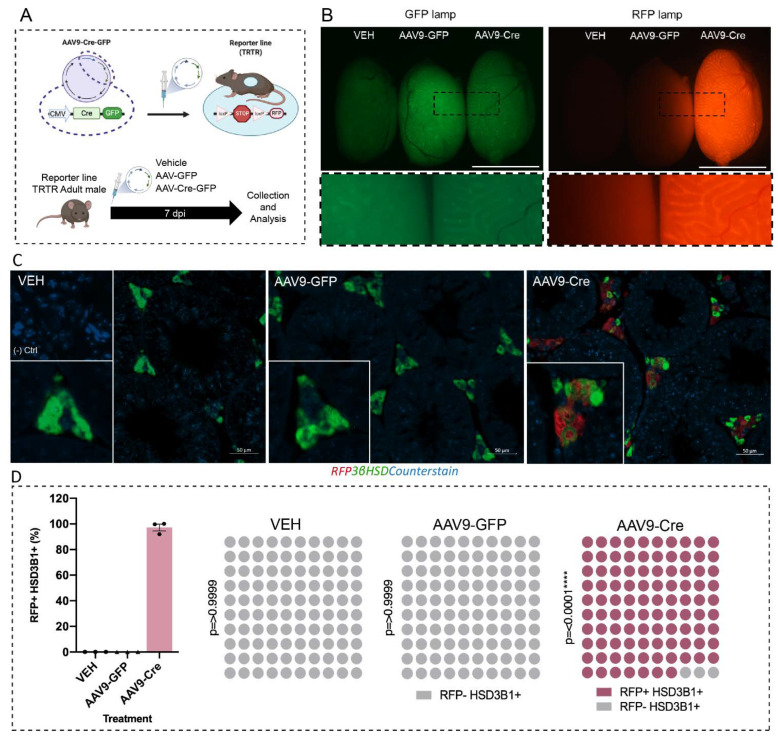
RFP expression in Leydig cells following delivery of the Cre Recombinase using adeno-associated virus serotype 9 (AAV9-Cre). (**A**) Schematic diagram of the generation of the inducible model (**B**) Representative images of freshly dissected Tomato Red (TRTR) testis at 7 days post injection (dpi) following injection of vehicle, AAV-GFP or AAV9-Cre-GFP; higher magnification of the dashed boxes is shown in the panel below. (**C**) Co-immunostaining of RFP and 3βHSD demonstrates RFP expression localised within Leydig cells (**D**) Quantification of cells co-expressing 3βHSD protein and RFP indicates only the AAV9-Cre-GFP induced expression of RFP in ~97% of Leydig cells (*n* = 3). (Chi~2 **** *p* = 0.0001). Scale Bars 100 μm.

We next investigated the physiological role of GR by acute ablation in adult Leydig cells (mice > 60 days of age) to dissect the effects of developmental vs. adult GR function in Leydig cells. We have previously demonstrated that AAV9 specifically and efficiently targets adult Leydig cells [30]. Thus, we utilised AAV9 carrying Cre recombinase to generate a GR knockout in adult Leydig cells (Figure 3A). 

First, to validate the targeting capability of AAV9-Cre in the adult testis, we used a Cre inducible Tomato Red Floxed reporter line (TRTR) [31] to verify the site of expression of the AAV9-Cre transgene (via expression of RFP). TRTR adult (postnatal day (d) 80) males were injected within the testicular interstitium with either vehicle, AAV9-GFP and AAV9-Cre-GFP (both carrying transgenes downstream of a CMV promoter) and the testes were collected 7 days post injection (dpi) (Figure 3A). The fluorescence was assessed on freshly dissected testis. While both AAV9-GFP and AAV9-Cre-GFP induced expression of GFP in the interstitium compared to the vehicle injected testis, only AAV9-Cre-GFP generated RFP expression within the testis (Figure 3B). To evaluate potential off-target effects following delivery of the Cre into the interstitial compartment, several organs were collected and assessed under for RFP expression. RFP was only detected in adrenal and liver (Supplementary Figure S3). To confirm that the downstream results are correlated with a direct consequence of GR depletion within the Leydig cell and not the off-target effects, we assessed the colocalization of GR and RFP proteins in both liver and adrenal. These results showed that cells expressing GR in the adrenal are not targeted by AAV9, suggesting that disruption of glucocorticoid signalling in the adrenal would be unlikely. There was a small degree of RFP and GR co-localisation observed in the liver (Supplementary Figure S4) but no changes in liver size, gross histology or health of animals were noted (not shown). We conclude that the AAV9-Cre-GFP is a suitable model to acutely target the testis with minimal off-target effects.

Next, to confirm that the AAV9-inducible Cre/*LoxP* system specifically targets Leydig cells within the testis, we co-localised the Leydig cell marker 3βHSD and RFP [32]. RFP localisation was restricted to 3βHSD-positive Leydig cells in the testis (Figure 3C). The number of RFP-positive versus RFP-negative Leydig cells was quantified in vehicle, AAV9-GFP and AAV9-Cre-GFP injected testes (7 dpi). In accordance with the immunohistochemistry (Figure 3C), only the AAV9-Cre-GFP induced expression of RFP in ~97% of Leydig cells (Figure 3D). These data demonstrate that AAV9 inducible Cre/*loxP* can target Leydig cells within 7dpi and thus is a suitable model for the inducible knockdown of GR expression in adult mice. 

### 2.4. Validation of the Inducible Model of GR Depletion in Adult Testis (AAV9-LCGR Mice)

**Figure 4:**
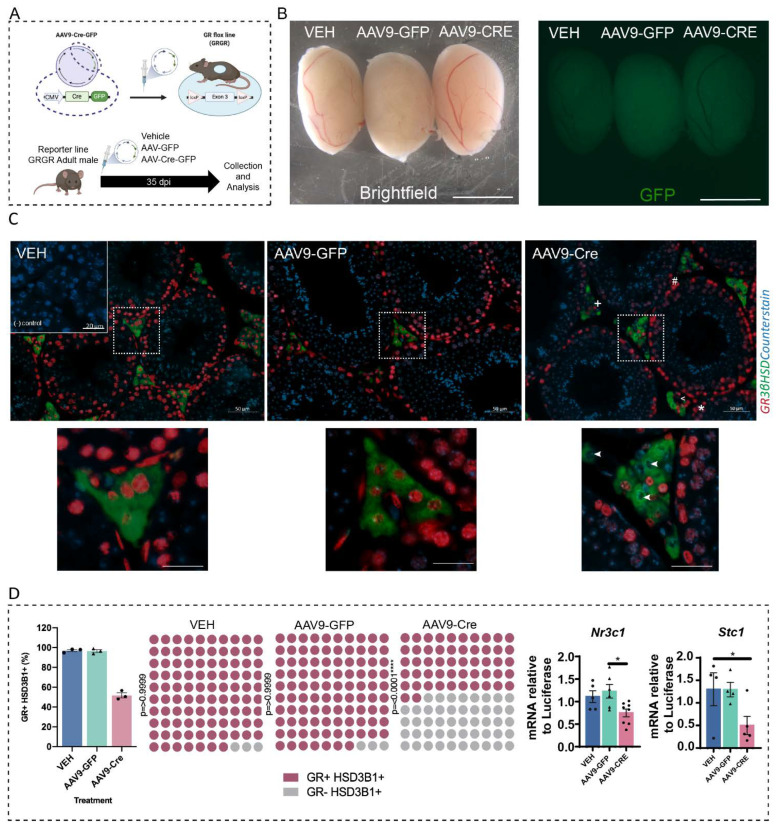
GR depletion in Leydig cells following delivery of the Cre Recombinase using AAV9-Cre-GFP. (**A**) Diagram of the model to ablate GR in adult Leydig cells (**B**) Representative fluorescent images of freshly dissected GRGR testis at 35 dpi injection of vehicle, AAV-GFP or AAV9-Cre. Scale bars 0.5 cm. (**C**) Co-immunostaining of GR and 3βHSD demonstrates depletion of GR protein in Leydig cells at 35 dpi AAV9-Cre. The micrographs in the bottom Insets demonstrate 40× magnification. Scale Bars = 50 μm. GR+ Sertoli cells, spermatogonia and spermatocytes (*), peritubular myoid cells (#), endothelial cells (<) and interstitial macrophages (+) are indicated. Arrowheads denote GR negative Leydig cells. (**D**) Quantification of cells co-expressing 3βHSD protein and GR indicates only the AAV9-Cre-GFP induced GR ablation, and this was observed in 48% of Leydig cells (*n* = 3, Chi-square **** *p* = 0.0001). Analysis of the testicular expression of Nr3c1 and Stc1 by qPCR demonstrates significantly reduced transcript levels (one-way ANOVA; *n* = 6–8, * *p* < 0.05, Tukey’s post hoc analysis, error bars SEM).

Adult (>d60) GR^flox/flox^ males (GRGR mice) (Figure 4A) were injected with either vehicle, AAV9-GFP or AAV9-Cre-GFP in their testicular interstitium and sacrificed 35 days later (the duration of mouse spermatogenesis) (Figure 4A). Fluorescence was assessed on freshly dissected testis and confirmed GFP expression in the interstitial compartment in both AAV9-GFP and AAV9-Cre-GFP compared the vehicle-injected testis (Figure 4B). Consistent with the inducible AAV9-Cre-GFP recombinase expression and action in the Tomato Red reporter line (Figure 3), GRGR mice injected with AAV9-Cre-GFP (hereafter referred to as AAV9-LCGR mice) displayed an absence of GR protein specifically in 3βHSD+ Leydig cells but not in other GR-expressing cells including Sertoli cells, germ cells, peritubular myoid, endothelial cells and interstitial macrophages (Figure 4C). Quantification of the number of 3βHSD-positive Leydig cells with or without GR expression revealed GR was depleted in ~48% of Leydig cells (Figure 4D). The increased number of GR-ablated Leydig cells in AAV9-LCGR mice, compared to the CYPGRKO mice, was reflected by decreased testicular expression of *Nr3c1* expression and of its direct downstream target, *Stc1* (Figure 4D), indicating that even a halving of the number of adult Leydig cells expressing GR leads to a reduced read out of GR-mediated transcription in the testis. 

In summary, we have developed a model that permits the acute and specific ablation of GR in approximately half of adult Leydig cells, allowing the investigation of the effects of reduced GR signalling in Leydig cells separate from developmental impacts. This model also provides a unique opportunity to determine the roles of autocrine GR signalling within Leydig cells, as has been previously shown for AR [28].

### 2.5. Reduced GR Signalling in Adult Leydig Cells Does Not Impact the Reproductive System

**Figure 5:**
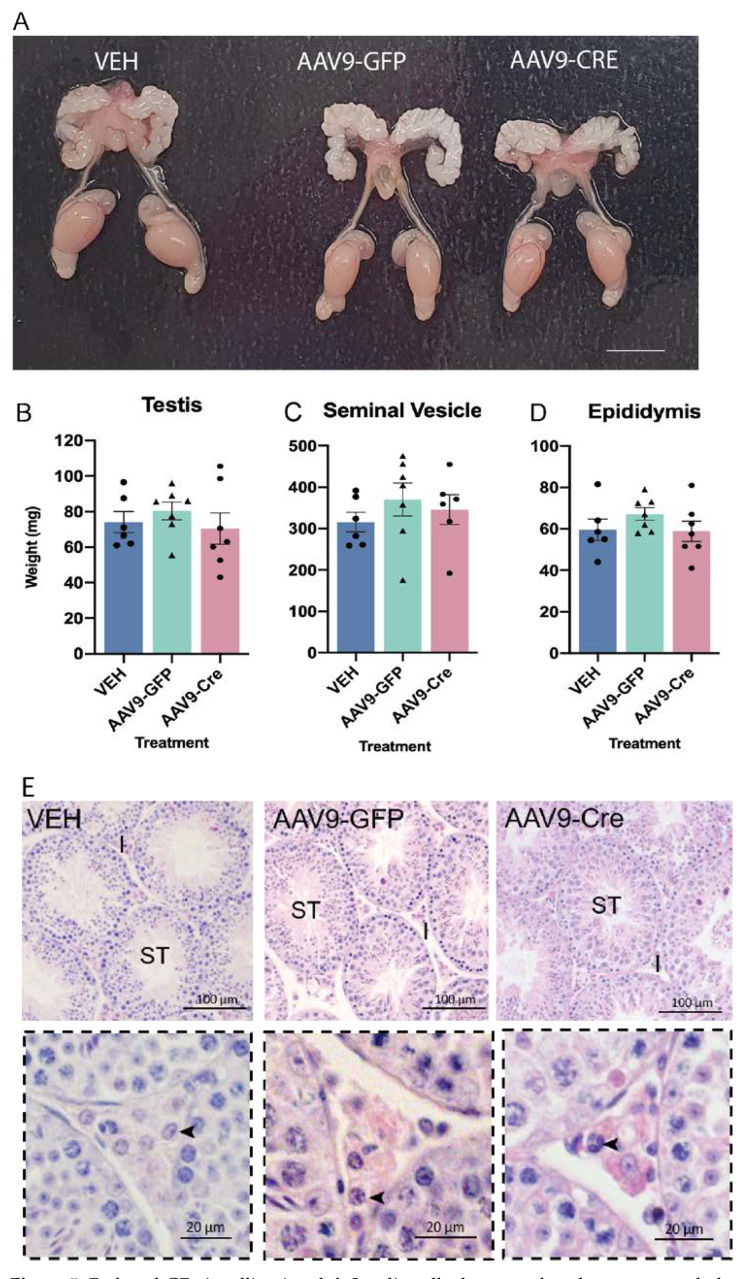
Reduced GR signalling in adult Leydig cells does not alter the gross morphology of the male reproductive system. (**A**) The gross morphology of the reproductive system in adult (d80) was comparable across the different groups. In adult (d80) no difference was noted in the (**B**) testis (**C**) seminal vesicles or (**D**) epididymis between the groups (*n* = 6–7, ANOVA). (**E**) Gross testicular morphology appeared normal. Insets in the panel below demonstrate 40× magnification of the testicular interstitium and arrowheads indicate the normal appearance of Leydig cells. Scale Bars = 100 μm, Abbreviations; I, Interstitium; ST, seminiferous tubule.

We assessed the impact of reduced GR signalling in AAV9-LCGR adult Leydig cells on the gross morphology of the reproductive system in males 35d post injection, however no differences were observed between the cohorts (Figure 5A). The body and reproductive organs (testis, seminal vesicle, and epididymis) weights between the cohorts were also unchanged (Figure 5B–D). As seminal vesicle weights serve as a key biomarker of circulating androgen action, this suggest that a reduction in GR signalling in adult Leydig cells does not have a major influence on circulating androgen levels. We next determined whether the loss of GR signalling in approximately half of the adult Leydig cells alters adult testicular architecture, however gross testis morphology in AAV9-LCGR mice was similar to controls (Figure 5E).

### 2.6. Reduced GR Signalling in Adult Leydig Cells Impairs Their Function

**Figure 6:**
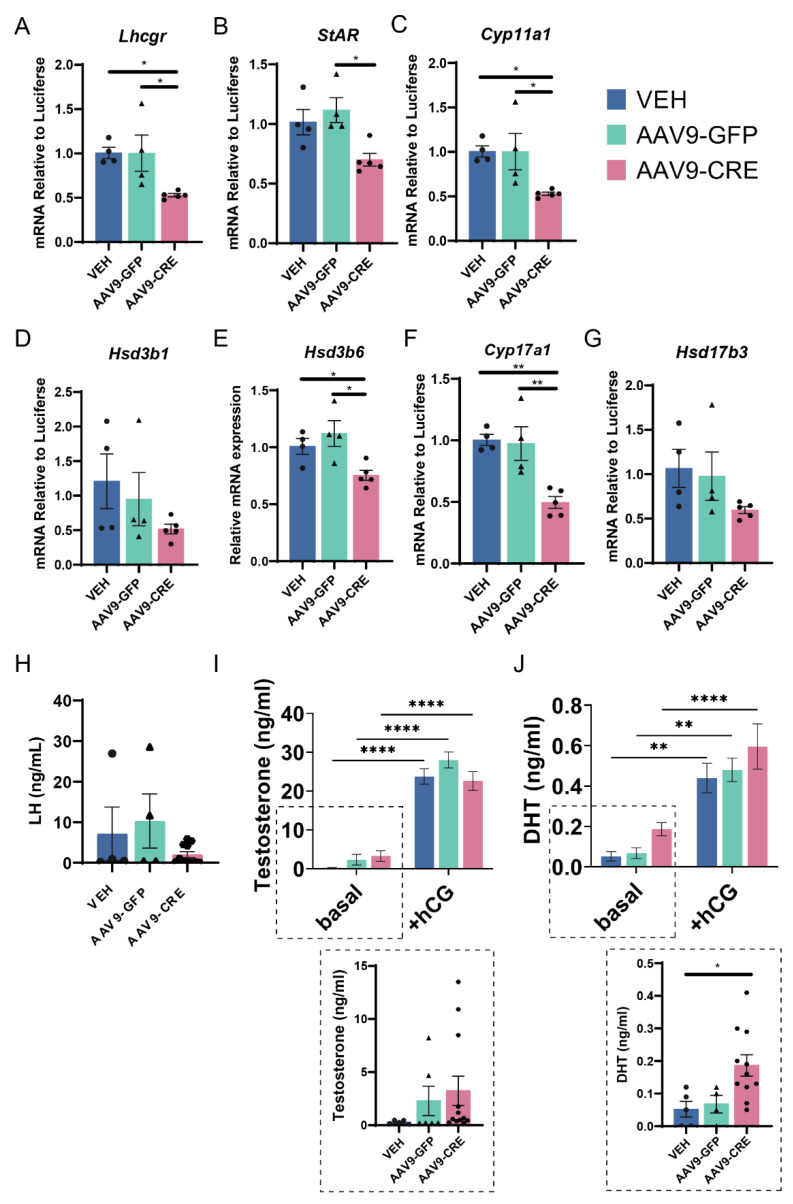
Reduced GR signalling impacts on Leydig cell function. (**A**–**I**) Comparative testicular expression of Leydig cell steroidogenic transcripts in adults (d80) in human chorionic gonadotrophin (hCG)-stimulated conditions (*n* = 5–7 per group ANOVA (* *p* < 0.05, ** *p* < 0.01 Tukey’s post hoc analysis, error bars SEM). (**H**) Circulating LH and circulating (**I**) testosterone, (**J**) DHT were assessed in basal and hCG-stimulated conditions. Insets show statistical analysis of testosterone and DHT in non hCG-stimulated mice, *n* = 5–7 per group ANOVA (* *p* < 0.05, ** *p* < 0.01 **** *p* < 0.0001, Tukey’s post hoc analysis, error bars SEM).

We further investigated the impact of reduced GR signalling on specific functional endpoints. Analysis of steroid enzyme transcripts and circulating hormones were carried out in uman Chorionic Gonadotrophin (hCG) stimulated mice. This is due to the intra-variability of the AAV9 targeting, and natural variation observed in Leydig cell function in mice, which is observed in unstimulated cohorts (Supplementary Figure S5). We stimulated Leydig cells of AAV9-LCGR and control mice), to induce maximum output from Leydig cells [28]. Steroidogenic enzymes transcripts (*StAR*, *Cyp11a1*, *Hsd3b6* and *Cyp17a1*) were significantly decreased (Figure 6A–F), however, the expression of *Hsd3b1* and *Hsd17b3*, the enzyme responsible for testosterone synthesis, was unchanged (Figure 6G). Circulating LH, and testosterone were not significantly altered (Figure 6H–I), though we do note an increase in serum DHT between AAV9-LCGR and vehicle-treated animals (Figure 6J), when analysed without the hCG-treated cohort; this could be physiologically important as DHT is a particularly potent androgen. Despite reduced levels of *Lhcgr*, there was no change in hCG-stimulated circulating androgen levels (Figure 6I,J). Taken together, the data suggest that a 50% reduction in GR signalling reduces Leydig cell expression level of key steroidogenic enzymes, indicating that GR signalling in Leydig cells supports their function.

## 3. Discussion

Glucocorticoids control fundamental processes in the human body including cell metabolism [33], growth [34], differentiation [35], apoptosis and inflammation [36]). Glucocorticoid signalling is a common target for pharmaceutical intervention in certain conditions such as in the treatment of chronic inflammation [37]) or anxiety and depression [38]) and GR agonists and antagonists are commonly used as treatments in a wide range of clinical settings. Given the widespread use of GR agonists and antagonists by males at different stages of the life course, it is important to determine the role of GR signalling in Leydig cell development and adult function, particularly in terms of steroidogenic capacity of the Leydig cells in adulthood. 

GR is expressed in the Leydig cells from fetal life onwards [15,39]. We first utilised the *Cyp17a1*-iCre model to investigate the role of GR in supporting the development and function of the adult Leydig cell population [26]. While the *Cyp17*-iCre model has previously been shown to efficiently target estrogen receptor alpha (*Esr1*) in Leydig cells, we found that only 28% of adult Leydig cells had depleted GR. Because this model could deplete *Nr3c1* expression in Leydig cells from late fetal life [26], it is possible that there could be some compensation during development that could lead to the majority of adult Leydig cells retaining GR. Depletion of GR signalling in Leydig cells from e16.5 encompasses the masculinisation programming window [26,40]. The normal development of the reproductive system in the adult CYPGRKO males suggests that the Leydig cells can maintain normal androgen levels across fetal life. The low level of GR ablation (28%) in adult CYPGRKO mice could be due to reduced targeting efficiency and/or developmental compensation during perinatal and pubertal Leydig cell proliferation and development. In adult CYPGRKO mice, ~70% of adult Leydig cells retain GR expression with no alterations in testicular morphology and steroid enzyme transcripts. demonstrating that the 70% of Leydig cells retaining GR expression can adequately compensate for any physiological loss of GR. This study is in in line with our previous androgen receptor knock out model in Leydig cells which has shown that targeting of approximately 50% is needed to have a substantial developmental and functional impact in Leydig cells [28]. The ability for nuclear receptors to be able to compensate for and activate reciprocal response elements, can add additional challenges when trying to define the role of a single nuclear receptor. Further consideration should be given to mineralocorticoid receptor, which has been shown to also have a high binding affinity for glucocorticoids and are present in Leydig cells [41], which may have also contributed to the lack of phenotype observed. Given the lack of suitability of the CYPGRKO model to effectively knockdown GR signalling in adult Leydig cells, and the lack of a phenotype in these mice, we next chose to develop a model of adult Leydig cell GR ablation.

We next focused on using a viral-mediated delivery system to knockdown Leydig cell GR signalling in adult mice. The recent characterisation of AAV-9 as tools to target Leydig cells provided an opportunity to develop an inducible Cre/loxP system [30] for this purpose. Utilising AAV-9. we generated a model that depletes GR in adult Leydig cells and found that this approach had improved targeting efficiency for Leydig cell gene knockdown compared to the Cyp17-iCre line. Furthermore, we demonstrated that this model had minimal off target GR ablation effects. Thus, our study offers the first evidence of a novel cost-effective method to investigate adult Leydig cell function and demonstrates that viral-mediated delivery of Cre recombinase to the interstitial compartment of the testis permits the knockdown of a gene of interest (in this instance GR) in adult Leydig cells. 

Use of the AAV9 system enabled refined assessment of the effects of reduced GR signalling in approximately half of the adult Leydig cells. It is known that supra- or sub-physiological levels of glucocorticoids can impact Leydig cell function and survival [21,42] and down-regulate the expression of *Lhcgr* [43] and steroidogenic transcripts [44]. However, there is little information regarding a requirement for GR-signalling in adult Leydig cell function. GR signalling in Sertoli cells is required to support normal testis development and Leydig cell differentiation and steroidogenesis [16]. Results from the present study demonstrate that GR signalling within Leydig cells is also important for Leydig cell steroidogenic function, as a significant decrease in Leydig cell steroidogenic enzyme expression was observed even when half of the Leydig cell population retained GR expression. We have previously shown that Sertoli cells support adult Leydig cell function and steroidogenesis [45] and thus the data from Hazra and colleagues [16] suggests that some of the Sertoli cell effects on Leydig cells are mediated by GR signalling in Sertoli cells. The mechanisms underlying the cross talk between Sertoli, and Leydig cell GR regulation warrants further investigation. 

Whilst our inducible Leydig cell knockout model did not impact the reproductive or testicular morphology, we observed a significant downregulation in steroidogenic enzyme transcripts, including *StAR, Cyp11a1*, and *Cyp17a1*. This suggests that GR-signalling is required to support Leydig cell steroidogenesis and confirms that GR has a physiological role across the somatic cells of the testis [16]. These variations were, however, not reflected in terms of circulating LH and testosterone levels, although DHT did show a significant increase. Taken together, the data suggests that depleting only 50% of GR signalling can induce a biological effect in Leydig cells, with a reduction in LHCGR expression and reduced transcription of key steroidogenic enzymes. The normal levels of circulating LH and testosterone may be associated with an ability of Leydig cells to maintain steroidogenic output in a setting of reduced enzyme expression [28] combined with the AAV9 targeting variations, across the cohorts, may explain the overall sampling variation. 

Both low and high levels of circulating glucocorticoids (as reported in Cushing Syndrome or Addison disease) suppress male steroidogenesis and fertility, whilst normal physiological levels regulate testis function [46,47]. This study developed two distinct Cre/*LoxP* models to target GR signalling in Leydig cells and showed that there is a threshold of GR inactivation to induce a Leydig cell defect (approximately 30% versus 50% GR targeting). This data highlights an intricate homeostatic balance of GR signalling in reproductive function, and whether it involves compensation mechanisms via the mineralocorticoid or other nuclear receptor remains to be investigated [48,49]. 

To conclude, our findings suggest that GR-signalling plays a physiological role in normal adult Leydig cell function. Our results also demonstrate the development of a novel transgenic mouse model that provides new opportunities to elucidate the roles of glucocorticoid signalling in Leydig cells, while simultaneously validating a new way to rapidly generate a model of adult Leydig cell gene knockdown. A considerable advantage of deliverable transgenics is that the panel of AAV isotypes permits targeting of different organs and cells, and thus our model is applicable to dissecting the genetic regulation of many physiological processes.

## 4. Materials and Methods

### 4.1. Ethics Statement

The research animals used in this study were monitored, handled, and euthanized in accordance with the NSW Animal Research Act 1998, NSW Animal Research Regulation 2010 and the Australian Code for the Care and Use of Animals for Scientific Purposes 8th Edition as approved by the University of Newcastle Animal Care and Ethics Committee (approval number A-2018-827 and A-2018-823). Mice used for experiments were housed at the institute’s Central Animal House under a 12 h light/12 h dark cycle at a constant temperature of 21–22 °C with food and water ad libitum. Animals were euthanized immediately before use via carbon dioxide asphyxiation.

### 4.2. Generation of CYPTR Reporter and CYPGRKO Knockout Mice Using Cyp17a1-iCre

To generate Leydig cell reporter mice, female C57BL/6 mice carrying a random insertion of the *Cyp17a1-i* Cre [50] were mated to C57BL/6 male mice homozygous (Hom) for a floxed *loxP*-flanked STOP cassette preventing transcription of a CAG promoter-driven red fluorescent protein variant (tdTomato) [31]. These matings resulted in Cre- Heterozygous (Het) ‘Controls’ and Cre+ Het ‘CYPTR’ mice. To generate Leydig cell GR knockout mice, female C57BL/6 mice carrying a random insertion of the *Cyp17a1-i* Cre [50] were mated to C57BL/6 male mice homozygous (Hom) for floxed GR [27]. The first generation resulted in offspring heterozygous (Het) for GRflox that were either Cre+ or Cre-. For total Leydig cell GR ablation Cre+ GR heterozygous males were again bred to C57BL/6 female mice homozygous for floxed GR resulting in the following offspring: Cre- Hom termed ‘littermate control’, Cre- Het, termed ‘Het littermate control’, Cre+ Hom, termed ‘CYP GR knockout’ (CYPGRKO), and Cre+ Het, termed ‘Het CYPGR knockout’ (Het CYPGRKO). 

### 4.3. PCR Genotyping of Mice

Mice were genotyped for the inheritance of Cre recombinase as previously described [51]. PCR amplification products were resolved using QIAxcel capillary system (QIAGEN, Sydney, NSW, Australia). An amplicon of 102 bp indicated the inheritance of the Cre recombinase transgene. Mice were also genotyped for the inheritance of floxed GR using primer sequences forward GGCATGCACATTACTGGCCTTCT, reverse 1 GTGTAGCAG CCAGCTTACAGGA and reverse 2 CCTTCTCATTCCATGTCAGCATGT. Expected band sizes are 2.5 kb for wild type GR and 500 bp for recombined GR. 

### 4.4. Viral Vectors

Adeno-Associated viral particle 9 (AAV9) containing GFP and CRE expressing transgenes downstream of a CMV promoter were obtained from GeneCopoeia (via United Bioresearch, Maroota NSW, Australia, Cat. No: AC001). Viral particles were supplied at a titre of ≥5 × 10^12^ GC/mL. Utilising viral vectors that express fluorescent reporters downstream of the powerful CMV promoter enabled confirmation and identification of all transduced cells carrying delivered transgenes (GFP and/or Cre recombinase). 

### 4.5. Testicular Delivery of Adeno-Associated Viral Particle 9

Viral particles were introduced into the interstitial compartment of adult (>60 days post-natal) mouse testes using an Ultra-Fine 23 gauge 0.3-mL insulin needle, injecting close to the rete testis as previously described [30]. A maximum volume of 20 μL was delivered. Successful delivery of the particles was monitored via the addition of Trypan Blue dye to the viral particles (0.04%). Animals were culled 7 days or 35 days post injection.

### 4.6. Inducible Model Using AAV9 Viral Vector

For assessment of the viral vector Cre recombinase delivery, we utilised Gt(ROSA)26Sor(tdTomato-WPRE) termed ‘TRTR’ mice carrying an insertion of a Cre reporter allele inserted into the Gt(ROSA)26Sor locus [31]. When TRTR mice are injected with the Cre-encoding viral construct directly into the interstitial area, RFP will be expressed specifically in cells where Cre was active. These mice were obtained from the Jackson laboratory (JAX stock #007914). To generate Leydig cell GR knockout mice, GR floxed (‘GRGR’, JAX stock #007909) mice which possess *loxP* sites flanking exon 3 of the *Nr3c1* gene, were injected as previously described. 

### 4.7. Tissue Collection and Processing

Mice were culled between d80 and d100 by inhalation of carbon dioxide and subsequent cervical dislocation Body weight was measured and organs were removed and weighed. Tissues were fixed in Bouin’s fixative for 4–24 h depending on tissue size. Bouin’s-fixed tissues were processed and embedded in paraffin wax, and 5 µm sections were used for histological analysis. Tissues were stained with haematoxylin and eosin using standard protocols and examined for histological abnormalities. 

### 4.8. Quantitative RT-PCR

RNA was obtained from frozen tissues using the RNeasy Mini extraction kit with RNase-free DNase on the column digestion kit (Qiagen, Sydney, NSW, Australia) according to the manufacturer’s protocol. RNA yield was quantified using a NanoDrop 1000 spectrophotometer (Thermo Fisher Scientific, Waltham, MA, USA). Random hexamer primed cDNA was prepared using the SuperScript VILO cDNA synthesis kit (Life Technologies) according to manufacturers’ protocols. Quantitative PCR was performed on the genes of interest listed in Table 1 using an Lightcycler 96 instrument (Roche, Sydney, NSW, Australia) and the Roche Universal Probe Library (Roche, AU). The expression of each gene was related to external housekeeping gene assay Luciferase (Roche, AU).

### 4.9. Immunohistochemistry

Immunolocalization was performed either by a single antibody colourimetric (DAB) immunostaining method, as described previously [52] a single or double antibody tyramide fluorescent immunostaining method, as described previously [50,53], or automated Bond immunostaining method, as described previously [52]. Antibodies used are listed in Table 2. A minimum of five individual sections for each genotype were immunostained in each experiment. For whole organ fluorescence, freshly dissected organs were visualised with a Zeiss Discovery V.12 Fluoroscope under either brightfield (transmitted light) or an epifluorescent GFP/RFP filter.

### 4.10. Extraction and Analysis of Steroid Hormones from Plasma

Immediately after culling (after CO_2_ and before cervical dislocation), blood was collected from mice via cardiac puncture with a syringe and needle with a wide bevel to reduce lysis, blood was collected in EDTA coated tubes to prevent coagulation. Plasma was separated by centrifugation and stored at −80 °C. LH was measured by ELISA according to manufacturer instructions (ab235648). The inter-assay coefficient of variance (CV) was <5.2% and the intra-assay CV was <5.4%. Steroid hormones were assessed using LC-MS/MS at the ANZAC Research Institute (University of Sydney, Sydney, NSW, Australia) as previously described [54].

### 4.11. Assessment of Cre Efficiency in Cre/LoxP and AAV9 Inducible Mice

Analysis of Cre efficiency in reporter mice and GR knockout mice was achieved via quantitation of cells positive for the Leydig cell specific protein 3βHSD and the reporter gene RFP, and the presence/absence of glucocorticoid receptor immunohistochemical localisation. Testis sections were imaged and then counted in Zen lite (ZEISS)). Each cohort had *n* = 3 and 5 sections from each animal was counted. Each section was scanned on the Axioscan to provide a whole section view. 

### 4.12. Statistical Analysis

Power calculations based on previous cell quantitation experiments determined that a sample size of 3 is appropriate for quantitative end points for cell counting and immunohistochemistry [55]. Statistical analysis is performed via GraphPad Prism (version 8; GraphPad Software Inc., San Diego, CA, USA). Statistical tests include a one-way ANOVA with Tukey’s post hoc test (if comparing multiple groups), a two-way ANOVA with Tukey’s post hoc test (if comparing multiple groups and variables), and Chi Squared test for determining proportion targeting/ablation. Values are expressed as mean ± S.E.M.

## Figures and Tables

**Table 1:** Details of Antibodies and detection methods used.

Primary Antibody (AbI) Name	References	Dilution AbI	RRID	Detection System
GFP	Abcam ab6556	1/1500	AB_305564	IHC/IF
3 b-HSD	Elabscience E-AB15112	1/1000	ND	IHC/IF
RFP	Evrogen #AB233	1/1500	AB_2571743	IHC/IF
GR	Abcam ab183127	1/1000	AB_2833234	IHC/IF

IHC: Immunohistochemistry IF: immunofluorescence.

**Table 2:** Details of primer sequences used for genotyping and qRT-PCR.

Gene	Forward Primer	Reverse Primer	Probe
*GRGR-FLOX*	Atgcctgctaggcaa atgat	Ttccagggctataggaagca	Genomic
*CYP17A1 ICRE*	CaggttttggtgcacagtCa	GctgtagcttctccactcCac	Genomic
TRTR WT	Aagggagctgcagtggagta	Ccgaaaatctgtgggaagtc	Genomic
TRTR MUTANT	Ggcattaaagcagcgtatcc	Ctgttcctgtacggcatgg	Genomic
*Lhcgr*	Gggacgacgctaatctcg	Cctggaaggtgccactgt	Upl #80
*Star*	Aaactcacttggctgctcagta	Tgcgataggacctggttgat	Upl #83
*Cyp11a1*	Cccattggggtcctgttta	Tggtagacagcattgatgaacc	Upl #67
*Cyp17a1*	Catcccacacaaggctaaca	Cagtgcccagagattgatga	Upl #67
*Hsd3b1*	Gaactgcaggaggtcagagc	Gcactgggcatccagaat	Upl #12
*Hsd3b6*	Accatccttccacagttctagc	Acagtgaccctggagatggt	Upl #95
*Hsd17b3*	Gagttggccagacatggact	Agcttccagtggtcctctca	Upl #47
*Srd5a1*	Gggaaactggatacaaaataccc	Ccacgagctccccaaaata	Upl #41
*Srd5a2*	Ggtcatctacaggatcccaca	Tcaataatctcgcccaggaa	Upl #50
*NR3C1*	Caaagattgcaggtatcctatgaa	Cttggctcttcagaccttcc	Upl #81
*STC1*	Gaggcggaacaaaatgattc	Gcagcgaaccacttcagc	Upl #45
*TSC22D3*	Ggtggccctagacaacaaga	Tcaagcagctcacgaatctg	Upl #10
*Luciferase*	Gcacatatcgaggtgaacatcac	Gccaaccgaacggacattt	5′ned- tacgcggaatacttc

## Data Availability

All data relating to this study has been included in the main manuscript or in the supplementary document. Schematics of breeding method Created with BioRender.com, agreement number BV24KRQ930.

## Supplementary Materials

The following supporting information can be downloaded at: https://www.mdpi.com/article/10.3390/ijms232315015/s1.
