# Supplementary material for: A Novel Model Using AAV9-Cre to Knockout Adult Leydig Cell Gene Expression Reveals a Physiological Role of Glucocorticoid Receptor Signalling in Leydig Cell Function
Source: Int J Mol Sci. 2022 Nov 30;23(23):15015. doi: 10.3390/ijms232315015 (PMC9737203; doi:10.3390/ijms232315015)
Supplement: Supplementary file 1 [file ijms-23-15015-s001.zip › ijms-2027666-supplementary.pdf]

A Novel Model Using AAV9-Cre to Knockout Adult Leydig Cell Gene Expression Reveals a Physiological Role of Glucocorticoid Receptor Signalling in Leydig Cell Function

Anne-Louise Gannon [1], Annalucia L. Darbey [1], Grace Chensee [1], Ben M. Lawrence [1], Liza O'Donnell [1], Joanna Kelso [1], Natalie Reed [1], Shanmathi Parameswaran [1], Sarah Smith [1], Lee B. Smith [1,2,3] and Diane Rebourcet [1,*]

[1] College of Engineering, Science and Environment, The University of Newcastle, Callaghan, NSW 2308, Australia
[2] MRC Centre for Reproductive Health, The Queen's Medical Research Institute, University of Edinburgh, 47 Little France Crescent, Edinburgh EH16 4TJ, UK
[3] Office for Research, Griffith University, Southport, QLD 4222, Australia
[*] Correspondence: diane.rebourcet@newcastle.edu.au

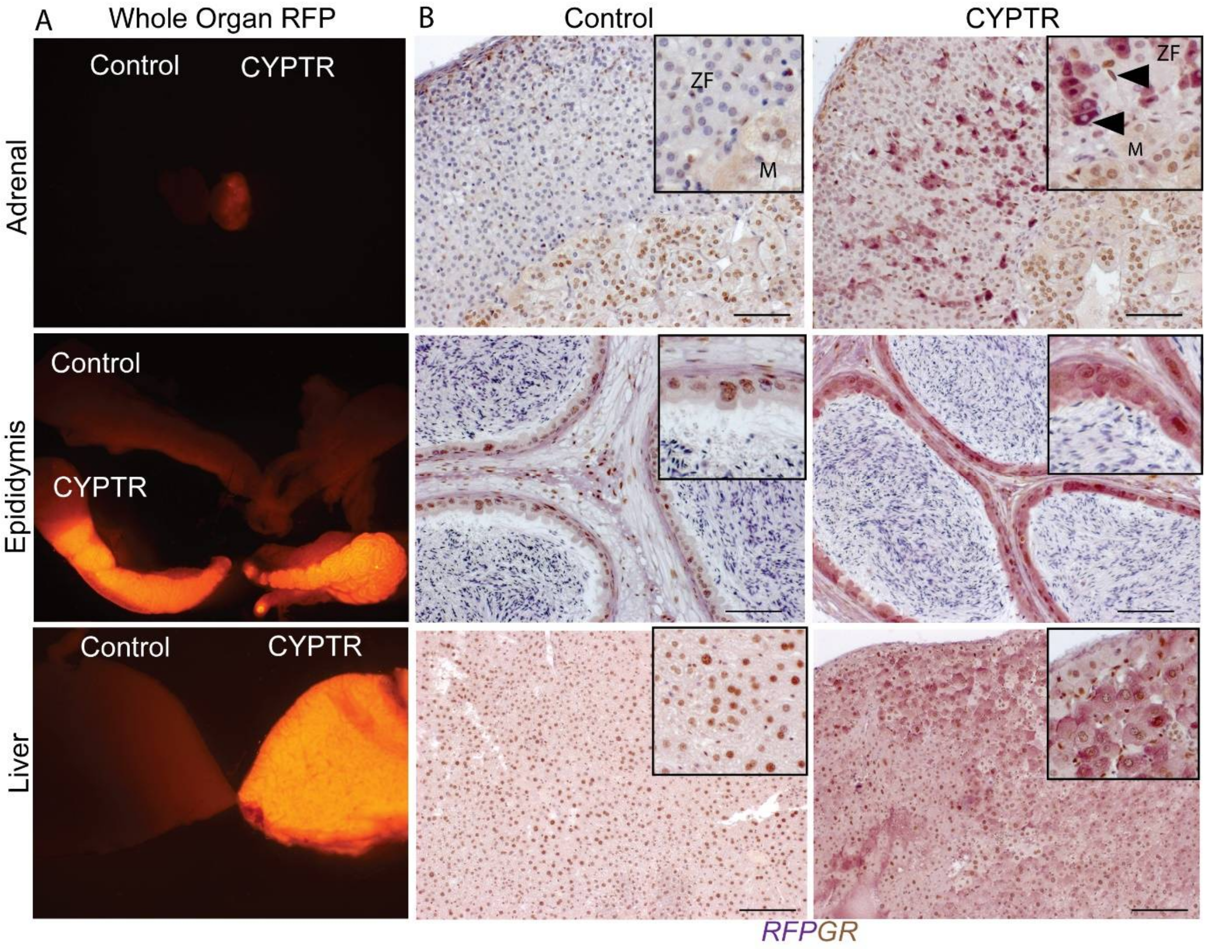

Supp Figure S1. Expression of RFP in tissues from *Cyp17a1*-iCre:RT26 RFP mice. (A) Representative images of freshly dissected CYPTR reproductive and non-reproductive tissues. Epifluorescence microscopy in adulthood shows expression of RFP in brain, pituitary, adrenal, epididymis and liver at d80. Tissues were imaged under an RFP filter demonstrating lack of RFP/autofluorescence in controls and positive RFP expression in CYPTR adrenal, epididymis and liver at d80 (fluorescent images taken after 5.5 seconds of exposure). **(B)** Double immunostaining of RFP and GR in tissue sections demonstrated no co-localisation with GR in adrenal cortex cells (denoted by arrow heads). Co-localisation is observed in the epithelial cells of the epididymis and hepatocytes of the liver. Insets demonstrate 40x magnification of single channel 3βHSD, RFP and merged channels. All mice were collected and analysed at d80. Scale Bars100 μm.

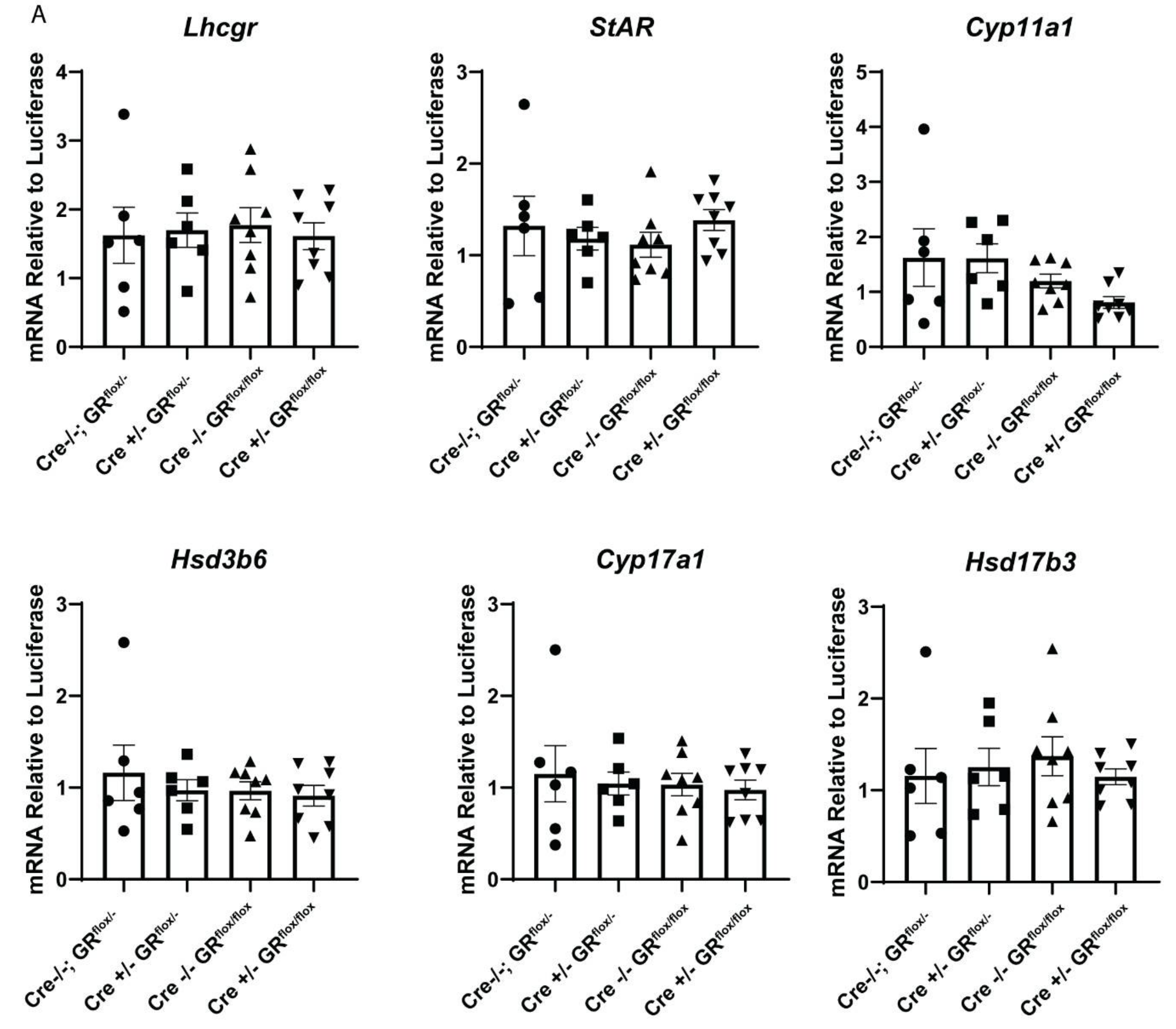

Supp Figure S2. CYPGRKO Leydig cells GR ablation does not disrupt steroid enzyme gene transcripts. (A) Analysis of testicular gene expression of key steroid enzyme transcripts *Lhcgr, StAR, Cy11a1, Hsd3b6, Cyp17a1 and Hsd17b3* reveals no changes in CYPGRKO males when compared with littermate controls (one-way ANOVA; n=6-8, Tukey's post-hoc analysis, error bars SEM).

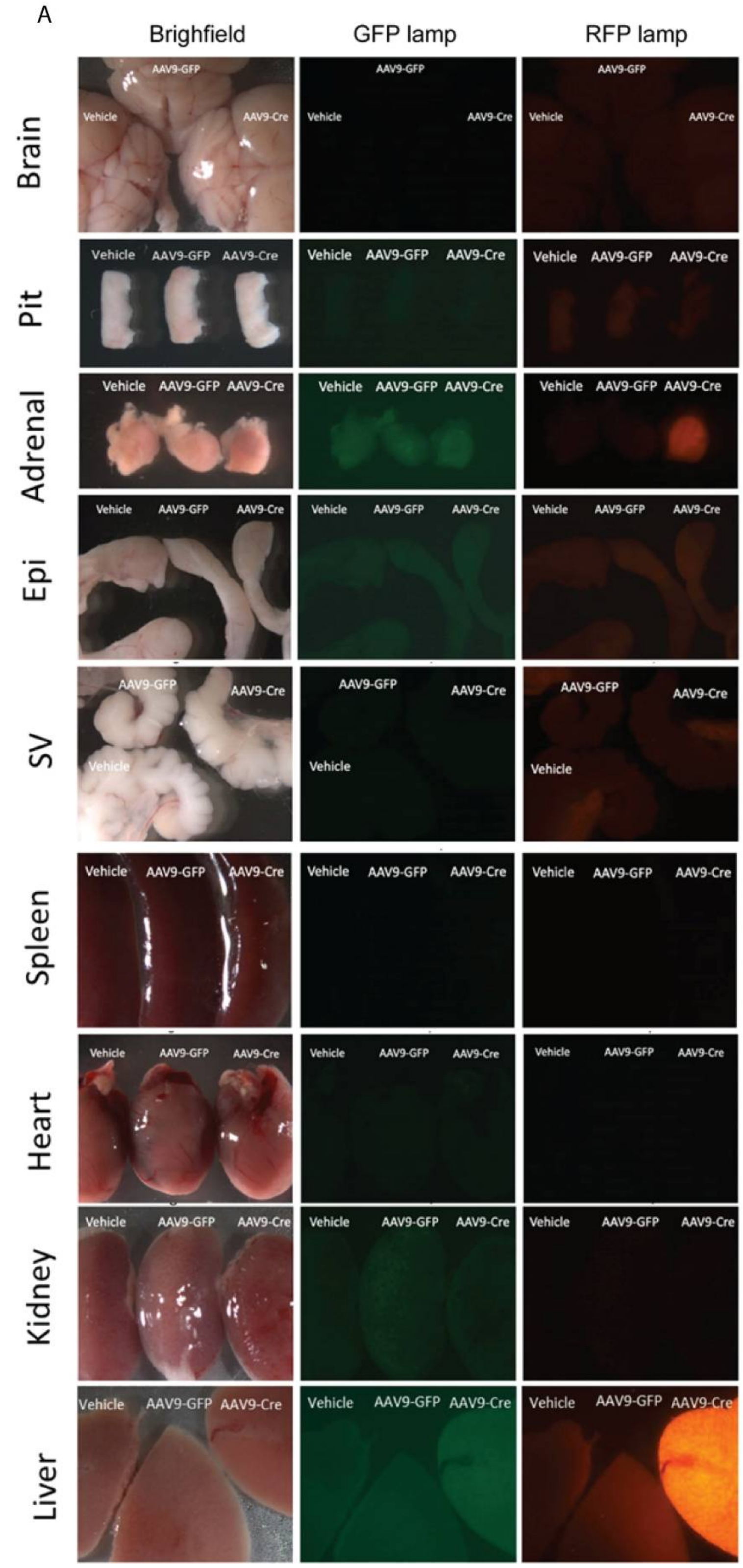

Supp Figure S3. Expression of RFP in tissues from AAV9-injected RT26 RFP mice. (A) Epifluorescence microscopy in adulthood shows expression of RFP in adrenal and liver in AAV9-injected TRTR mice. Tissues were imaged under an RFP filter demonstrating lack of RFP/autofluorescence in controls and positive RFP expression in AAV9-Cre adrenals and liver (fluorescent images taken after 5.5 seconds of exposure).

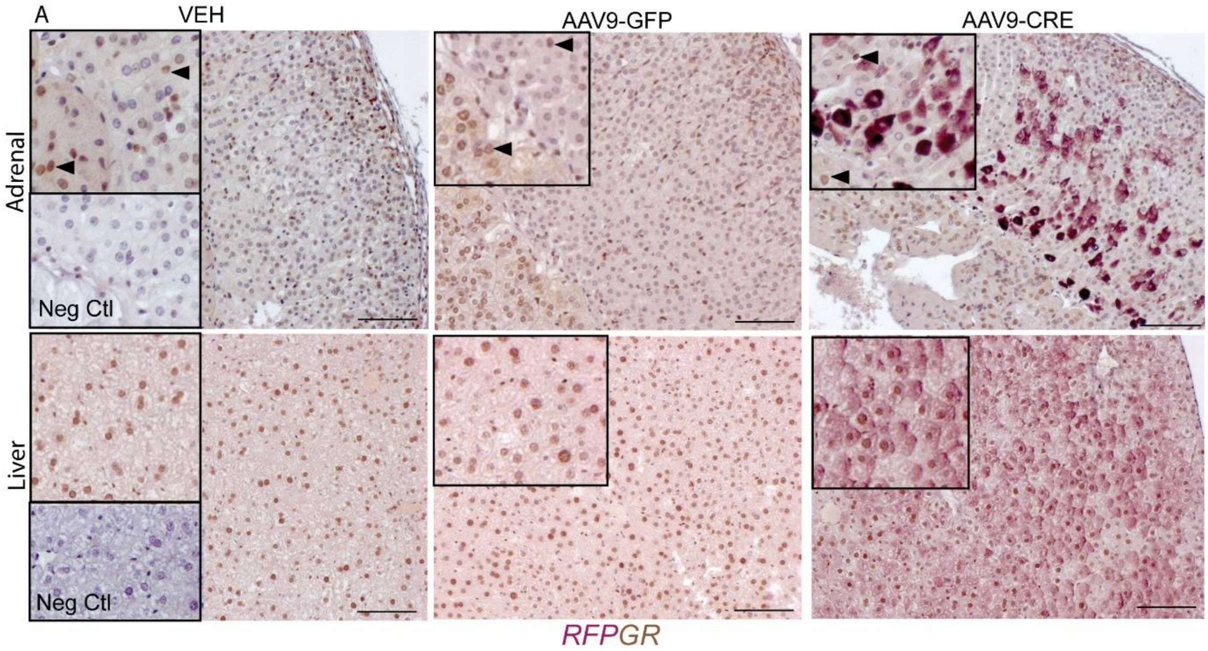

Supp Figure S4. GR does not co-localise with RFP expression in the adrenal cortex. (A) Double immunostaining of RFP tissue sections demonstrated no co-localisation with GR in adrenal cortex cells (denoted by arrow heads). Co-localisation is observed in the hepatocytes of the liver. Insets demonstrate 40x magnification. Scale Bars 100 µm.

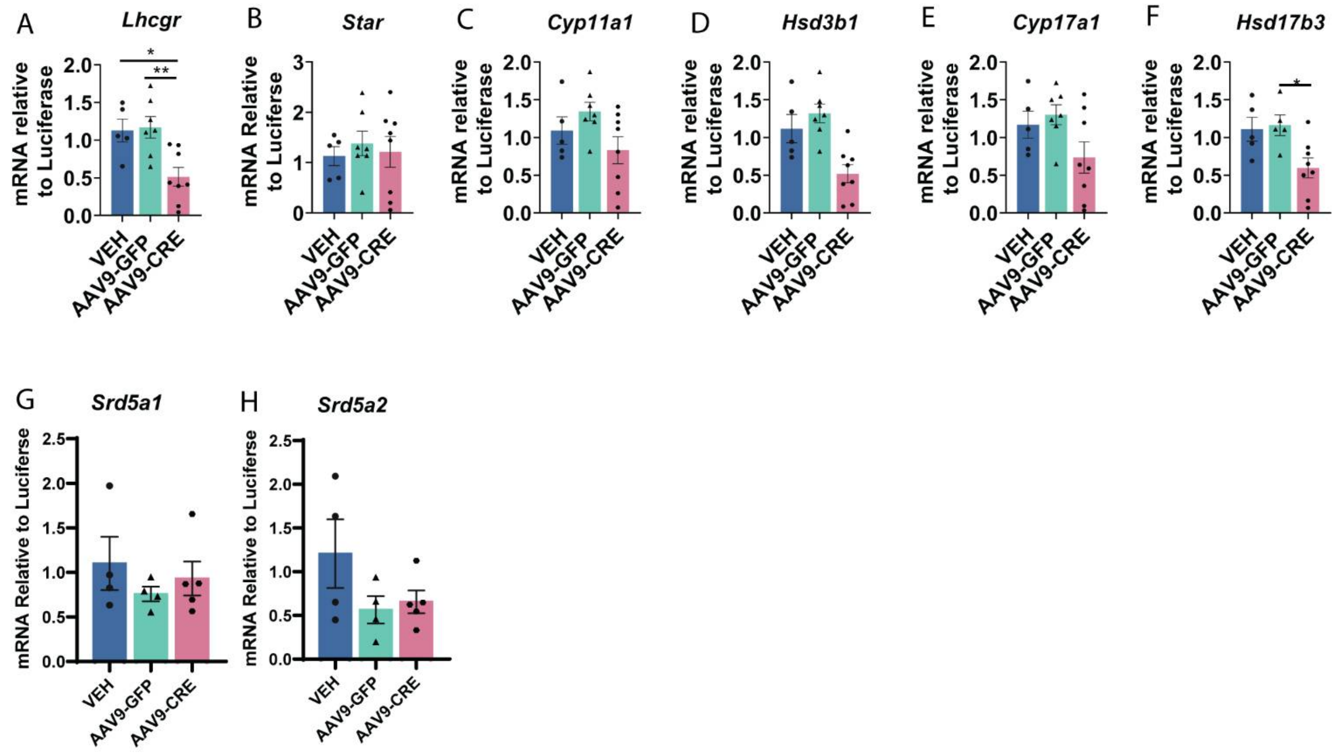

Supp Figure S5. Steroid enzyme gene transcripts in AAV9-Cre-GFP mice. (A-F) Comparative testicular expression of Leydig cell steroidogenic transcripts in adults (d80) in basal conditions (n = 6-8 per group, one-way ANOVA; n=6-8,* P < .05, **P < .01 Tukey's post-hoc analysis, error bars SEM). **(G-H)** Comparative testicular expression of androgen pathway genes *Srd5a1* and *Srd5a2*, show no changes following GR ablation (one-way ANOVA; n=6-8, * P < .05, **P < .01 Tukey's post-hoc analysis, error bars SEM).
